# An efflux-susceptible antibiotic-adjuvant with systemic efficacy against mouse infections

**DOI:** 10.1038/s41598-022-21526-4

**Published:** 2022-10-21

**Authors:** Ohad Meir, Fadia Zaknoon, Amram Mor

**Affiliations:** Faculty of Biotechnology and Food Engineering, Technion – Israel Institute of Technology, 3200003 Haifa, Israel

**Keywords:** Peptides, Biomimetics, Antimicrobials, Bacteria, Pathogens, Drug discovery and development

## Abstract

Scarcity of effective treatments against sepsis is daunting, especially under the contemporary standpoints on antibiotics resistance, entailing the development of alternative treatment strategies. Here, we describe the design and antibiotic adjuvant properties of a new lipopeptide-like pentamer, decanoyl-bis.diaminobutyrate-aminododecanoyl-diaminobutyrate-amide (C_10_BBc_12_B), whose sub-maximal tolerated doses combinations with inefficient antibiotics demonstrated systemic efficacies in murine models of peritonitis-sepsis and urinary-tract infections. Attempts to shed light into the mechanism of action using membrane-active fluorescent probes, suggest outer-membrane interactions to dominate the pentamer’s adjuvant properties, which were not associated with typical inner-membrane damages or with delayed bacterial growth. Yet, checkerboard titrations with low micromolar concentrations of C_10_BBc_12_B exhibited unprecedented capacities in potentiation of hydrophobic antibiotics towards Gram-negative ESKAPE pathogens, with an apparent low propensity for prompting resistance to the antibiotics. Assessment of the pentamer’s potentiating activities upon efflux inhibition incites submission of a hitherto unreported, probable action mechanism implicating the pentamer’s *de-facto* capacity to hijack bacterial efflux pumps for boosting its adjuvant activity through repetitive steps including outer-membrane adhesion, translocation and subsequent expulsion.

## Introduction

Sepsis represents one of the most notorious yet ill-treated medical conditions^1,2^, annually affecting 49 million patients worldwide, with a towering mortality rate of 20%^3,4^. Effective treatments are urgently needed as current strategies are limited to short-term immunomodulation^5–7^ and broad-spectrum antibiotics administration^8–10^, both of which do not adequately meet the challenge, hence allowing sepsis to persist as leading cause of death^3,4^. Development of effective treatments is complicated, as they must overcome multiple obstacles pertaining to treatment onset, pathogens diversity, as well as variation in host response^1,2^. This problem is exacerbated by increased prevalence of antibiotic resistance that further limits current treatments efficacies, particularly with regards to Gram-negative bacteria (GNB), against which, no new antibiotic classes have been successfully developed for over 50 years^11^. Consequently, exploration of antibiotics combinations is frequently pursued by clinicians, sometimes based on trial and error. Alternatively, immune curbing by agents indifferent to pathogens and/or host variability, may offer a more versatile and potentially preferred treatment strategy. Thus, the ability to sequester contributing factors, such as lipopolysaccharides (LPS) that promote sepsis deterioration to septic-shock, would represent a desirable attribute of such agents.

Cationic antimicrobial peptides (AMPs) are sometimes considered as potential candidates for accomplishing this task. AMPs chemo-physical attributes were evolutionarily designed to selectively target bacterial membranes, including through interactions with anionic moieties of LPS, such as lipid A^12–14^. Nevertheless, their clinical utilization is deemed challenging, namely due to their relatively short half-lives and host-toxicity upon systemic treatments, whereas peptidomimetic approaches are currently believed to minimize such drawbacks, imparting them with robustness and improved chances for successful clinical implementation^15^. Albeit, those characterized with membranolytic modes of action might in fact achieve the exact opposite outcome, as they too—appear to instigate unregulated LPS release. In this respect, antibiotic adjuvants (namely those using non-specific mechanisms) may provide an attractive treatment alternative^16^, as they can reinstate an antibiotic’s efficacy as well as reduce high-dosage associated toxicity^17^. Adjuvants can also bestow potency upon Gram-positive-specific hydrophobic antibiotics towards GNB^18^, specifically when resistance emanates from their low translocation across the outer-membrane (OM)^19^. Moreover, owing to their mechanistic distinctions from antibiotics, adjuvants can be impervious to the documented resistance mechanisms, emphasizing an inherent advantage for their preferable utilization^20^. One such group of AMP-mimetics is represented by short lipopeptide-like sequences, composed of amide-linked fatty acids and cationic amino acids^21^, which, owing to their simple yet modular structure, have recently shown considerable aptitude to facilitate fine-tuning of the chemo-physical properties and subsequent AMP-like biological attributes^22–24^. Here, we sought out to investigate additional new analogs designed to function as adjuvants, through substitution of the cationic amino-acids and then attempted to elucidate the mechanism of action of the most promising emerging analog.

## Results and discussion

### C_10_BBc_12_B emerges as promising antibiotics adjuvant

**Figure 1:**
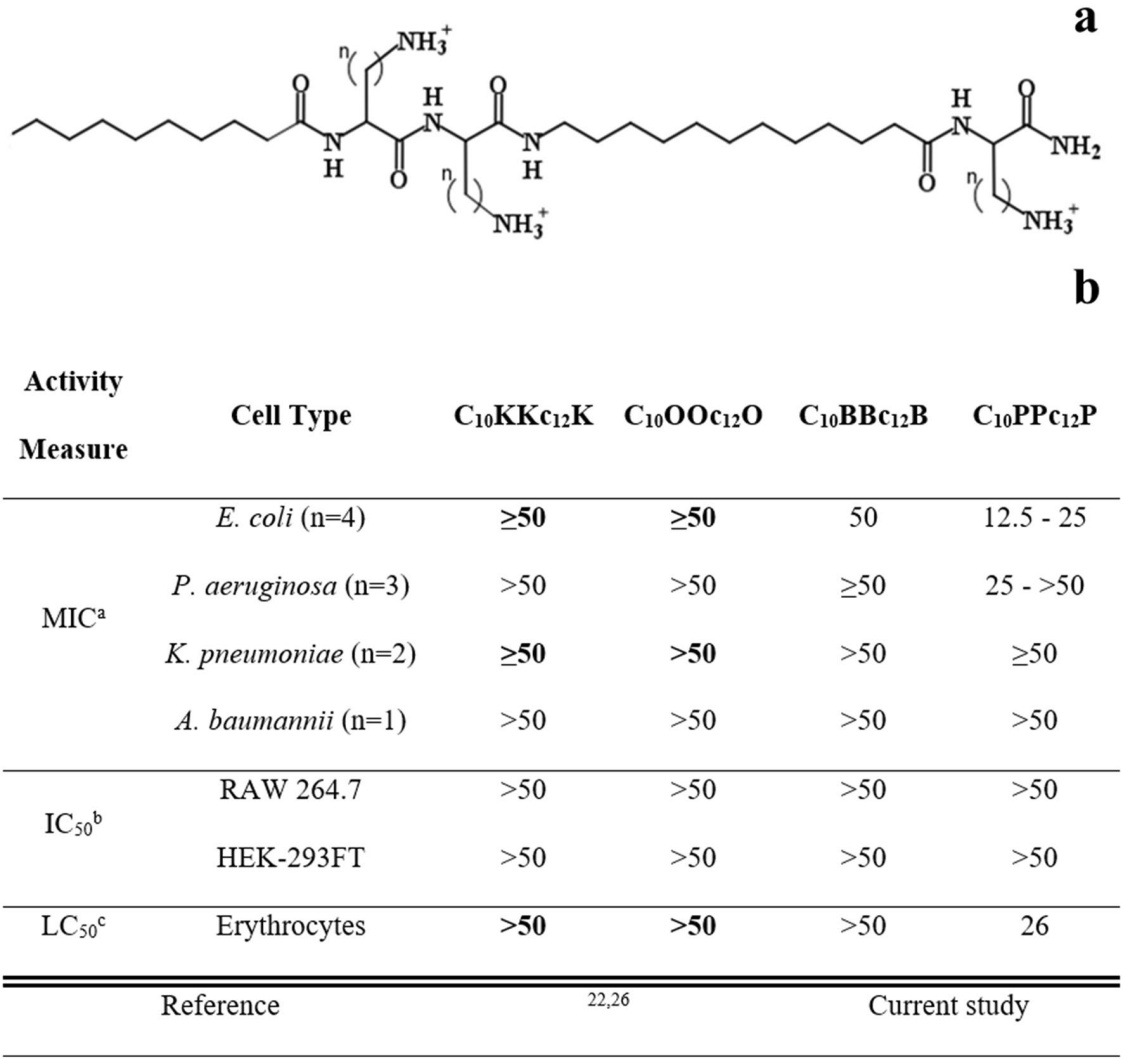
Basic Characterization of Pentamer Analogs. (**a**) General molecular structure of the A_10_CCa_12_C series; where A_10_ and a_12_ stand for decanoyl and aminododecanoyl, whereas “C” represents one of the following cationic amino acids: lysine (K), ornithine (O), diaminobutyric acid (B) or diaminopropionic acid (P) where n (methyl groups number on each side chain) equals 4, 3, 2 or 1, respectively. Molecular weights of these analogs are 753, 711, 669 and 627 g/mol, for K, O, B and P, respectively. (**b**) Cytotoxic activity of pentamer analogs. ^a^, Minimal inhibitory concentrations determined using the broth microdilution method (n, number of tested strains); ^b^, Pentamer concentration causing 50% inhibition of cells respiration, using Alamar blue; ^c^, Pentamer concentration causing 50% hemolysis of murine RBC. Published data appear in bold fonts, shown for comparison purposes.

Our previous attempts to design antibiotic adjuvants have focused on short lipopeptide-like sequences corresponding to the pentameric formula A_x_CCa_y_C; where “A_x_” and “a_y_”, respectively represent the N-terminal acyl of length x (number of methyl groups) and an aminoacyl of length y, whereas “C” represents a cationic amino acid. Based on earlier findings^24,25^, we abstained in the current study from incorporating an exceedingly hydrophobic acyl at the N-terminus, that could generate potent but non-selective antimicrobial lipopeptides, which in turn, might complicate their *in-vivo* systemic implementation due to toxicity phenomena such as hemolysis and unregulated LPS release. Instead, we used decanoic acid, which emerged from an analogous study^26^ as potentially embodying the appropriate hydrophobicity for the sequences under present investigation. In parallel, we also attempted to optimize contributions emanating from the cationic residues, by adjusting their side-chain length (Fig. 1a).

We first attempted to detect hydrophobicity differences among the cationic analogs by comparing their elution properties, using a C_18_ reversed-phase HPLC column. However, they all eluted between 45 and 46% hydrophobic solvent (acetonitrile), including upon co-injection of the four analogs, suggesting that the side-chains contribute very little to these molecules chromatographic behavior. This contrasted with previous observations where reducing or increasing the N-terminal acyl length by only 2 methylenes (e.g., C_12_KKc_12_K → C_10_KKc_12_K → C_8_KKc_12_K)^24^ resulted in substantial hydrophobicity difference (as deduced from the fact that their elution required approximately 5% reduced or increased acetonitrile concentration, respectively). Additionally, assessment of self-assembly tendencies of these lipopeptide analogs (through measuring their light scattering properties) has also demonstrated little dissimilarities in their critical aggregation concentrations, which were all ≥ 100 µM.

Conversely, evaluation of the lipopeptides bioactive properties revealed similarities only between K-, O- and B-based analogs (Fig. 1b), whereas the P-based pentamer presented a divergent activity profile. Namely, C_10_PPc_12_P exhibited a hemolytic capacity somewhat more pronounced than that observed with its analogs (*i.e.*, LC_50_ = 26 *versus* > 100 µM, respectively). Additionally, when tested against 10 bacterial strains representing the four species of Gram-negative ESKAPE pathogens, the K-, O- and B-based analogs displayed high minimal inhibitory concentrations (MIC ≥ 50 µM), whereas C_10_PPc_12_P occasionally presented somewhat lower MIC values. Notably, C_10_PPc_12_P presented a lower selectivity profile (i.e., its MIC and LC_50_ values were comparably scaled) which may be attributed to its side-chain’s amine pKa, as it is less likely to be fully protonated at neutral pH (i.e., its β-NH_2_ group pKa is 6.3^27^ *versus* at least 10.4^28^, for the three analogs). This could (at least partly) explain its similar potency against RBCs and bacterial cells, particularly since selectivity towards bacterial membranes is significantly charge-driven. Notably, no cytotoxicity towards cell-lines was observed at-least up to 50 µM.

Having established these analogs overall rather poor antibiotic activity against GNB, we next defined their capacities to function as adjuvants that sensitize bacteria to antibiotics. For this purpose, we determined the analogs ability, at a sub-inhibitory concentration (10 µM) to potentiate erythromycin and rifampin (representing two antibiotic families that are normally ineffective against GNB, namely due to their hydrophobicity) using *E. coli* as representative species. As shown in Table 1, all four analogs presented substantial potentiation capacities, although generally, potency appeared to increase as the cations transitioned from K to B and then decreased with P. Of note, despite the weaker growth-inhibitory activity of C_10_BBc_12_B (Fig. 1b), this pentamer exhibited the strongest potentiating activities (Table 1). This is namely evidenced by its lower calculated fractional inhibitory concentration index, compared with the next most potent analog (i.e., 0.2 *versus* 0.4, respectively for C_10_BBc_12_B and C_10_PPc_12_P).

**Table 1:** *E. coli* sensitization to antibiotics by adjuvants.

Antibiotic	Sensitization Factor (SF) in presence of 10 µM adjuvant				
	C_10_KKc_12_K	C_10_OOc_12_O	C_10_BBc_12_B	C_10_PPc_12_P	SPR741
Erythromycin	128 (4)	**512 **(32)	1024 (256)	512 (128)	NA **(1024)**
Rifampin	4096 (32)	**8192 **(256)	65,536 (8192)	32,768 (1024)	NA **(8192)**
Reference	Current study	^29^	Current study		^30^

Values specify bacterial sensitization factors (*i.e.*, fold difference between the antibiotic’s MIC in absence of adjuvant *versus* in its presence) to each antibiotic, as determined against *E. coli* 25922. SF values obtained for cyclic analogs are shown in parenthesis. Erythromycin and rifampin MIC values in absence of adjuvant were 128 and 8 µg/ml, respectively. Published data appear in bold fonts.

*NA¸* not applicable.

Since cyclization of AMPs was found to improve their potency, we similarly produced and tested the corresponding head-to-tail cyclic analogs of the four pentamers. As depicted in Table 1, while maintaining considerable potencies, the cyclic analogs presented substantially lower SF values than their linear counterparts. This finding suggests that structural rigidity contributes to greater antimicrobial potency, whereas flexibility appears to promote stronger adjuvant properties.

Table 1 also compares the pentamers sensitization factors (SF) values with those reported for SPR741, a promising cyclic polymyxin B nonapeptide derivative^31^ (PMBN) under clinical investigation^32^. Interestingly, when assessed in another study^30^ for its capacities to potentiate various antibiotics against 3 GNB species, SPR741 revealed SF values seldom exceeding 1000 folds which, in the case of erythromycin, we found quite similar to those of C_10_BBc_12_B (using the same *E. coli* strain). In the case of rifampin however, although both adjuvants displayed even higher SF values, C_10_BBc_12_B revealed to be substantially more potent.

**Figure 2:**
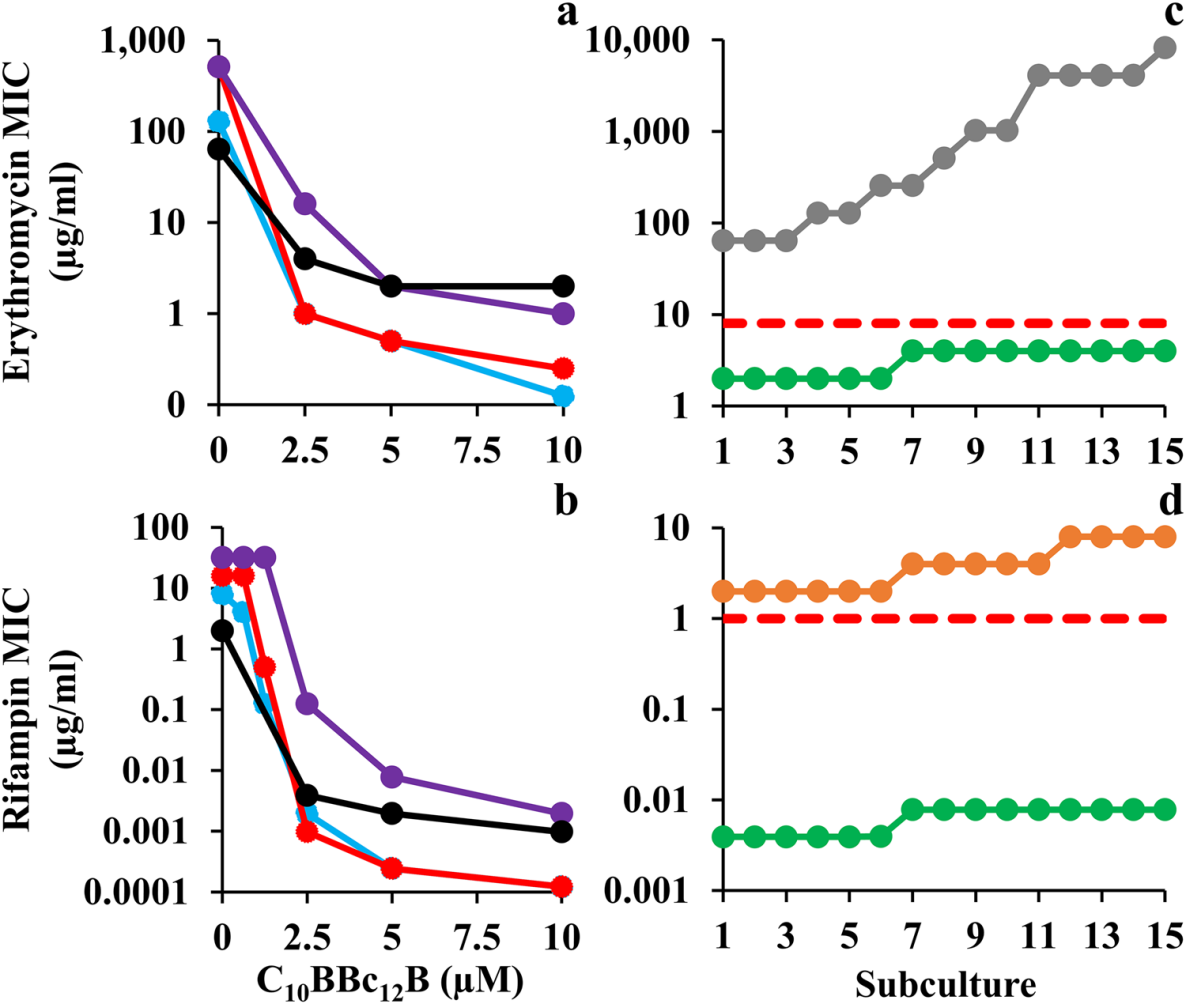
Sensitization of Gram-Negative ESKAPE Pathogens to Antibiotics. (**a**, **b**) Isobolograms generated for inhibitory combinations of C_10_BBc_12_B with erythromycin and rifampin, respectively. *E. coli* 25922, cyan; *K. pneumoniae* 1287, red; *P. aeruginosa* 27853, purple; *A. baumannii* 19606, black. (**c**, **d**) Antibiotic MIC evolution over 15 consecutive subcultures of *A. baumannii* 19606, as tested in duplicates, in absence *versus* in presence of 5 µM C_10_BBc_12_B. Erythromycin, gray; rifampin, orange; C_10_BBc_12_B, green; resistance breakpoint, dashed red lines.

A broader scope of C_10_BBc_12_B potentiation capacities is shown in Fig. 2, indicating that the pentamer is endowed with a similarly exquisite capability for sensitizing all four Gram-negative representatives of ESKAPE pathogens tested, as observed with both erythromycin and rifampin. The isobolograms (presented in panels a and b) display geometries indicative of definite synergistic relationships and in most cases, C_10_BBc_12_B has potentiated the antibiotics to a point requiring concentrations well-below their respective resistance breakpoints (i.e., 8 and 1 µg/ml respectively for erythromycin and rifampin, as defined against *Staphylococci*)^33^. For example, the pentamer reduced erythromycin’s inhibitory concentrations against *E. coli* and *K. pneumoniae* from 128 and 512 µg/ml to 0.125 and 0.250 µg/ml (i.e., SF values of 1024 and 2048, respectively). Likewise, rifampin’s MIC against these species was reduced to below 1 ng/ml, reflecting an increased sensitization efficiency of 65,000 and 130,000 folds, respectively. Such unprecedented values strengthen the proposed stature of C_10_BBc_12_B as an exceptionally potent antibiotic adjuvant.

### Pathogens sensitization is dissociated from emergence of resistance

To assess selective pressure effects on the observed bacterial sensitization to antibiotics, we compared the rates of antibiotic-resistance development by *A. baumannii* which occupies the top of the urgent category in the latest CDC’s antibiotics resistance threats report^34^. Panels c and d show that in presence of C_10_BBc_12_B, the MIC of either antibiotic has not increased by more than 2 folds, which corresponds to the inherent variance of the microdilution technique used during the subculture passages and thus can be regarded as practically unaltered. Conversely, in absence of C_10_BBc_12_B, antibiotics’ MIC values have increased by 128 and 4 folds, respectively for erythromycin and rifampin (i.e., from 64 to 8,192 µg/ml and from 2 to 8 µg/ml, respectively). This dissimilar increase maybe ascribed to the antibiotics differential modes of action (i.e., bacteriostatic *versus* bactericidal)^35^ or to their respective predominant resistance mechanism (i.e., efflux pumps over-expression^36^ or *rpoB* mutations^37^). Regardless, the fact that in presence of C_10_BBc_12_B the antibiotics’ MIC remained effectively constant, implies that the pentamer has effectively bypassed bacterial aptitudes for developing antibiotic-resistance (at least throughout the assay duration). While the molecular basis is yet to be elucidated, it is worth mentioning that similar assessment was recently reported for SPR741^32^ on *E. coli*^38^. Moreover, as despite the pentamer’s greater synergistic potency and the consequently greater evolutionary pressure exerted on resistance development^39^, resistance has not materialized, thereby highlighting potential advantages of C_10_BBc_12_B-based therapies.

### C_10_BBc_12_B performs as adjuvant ***In-Vivo*** to protect mice from infections

**Figure 3:**
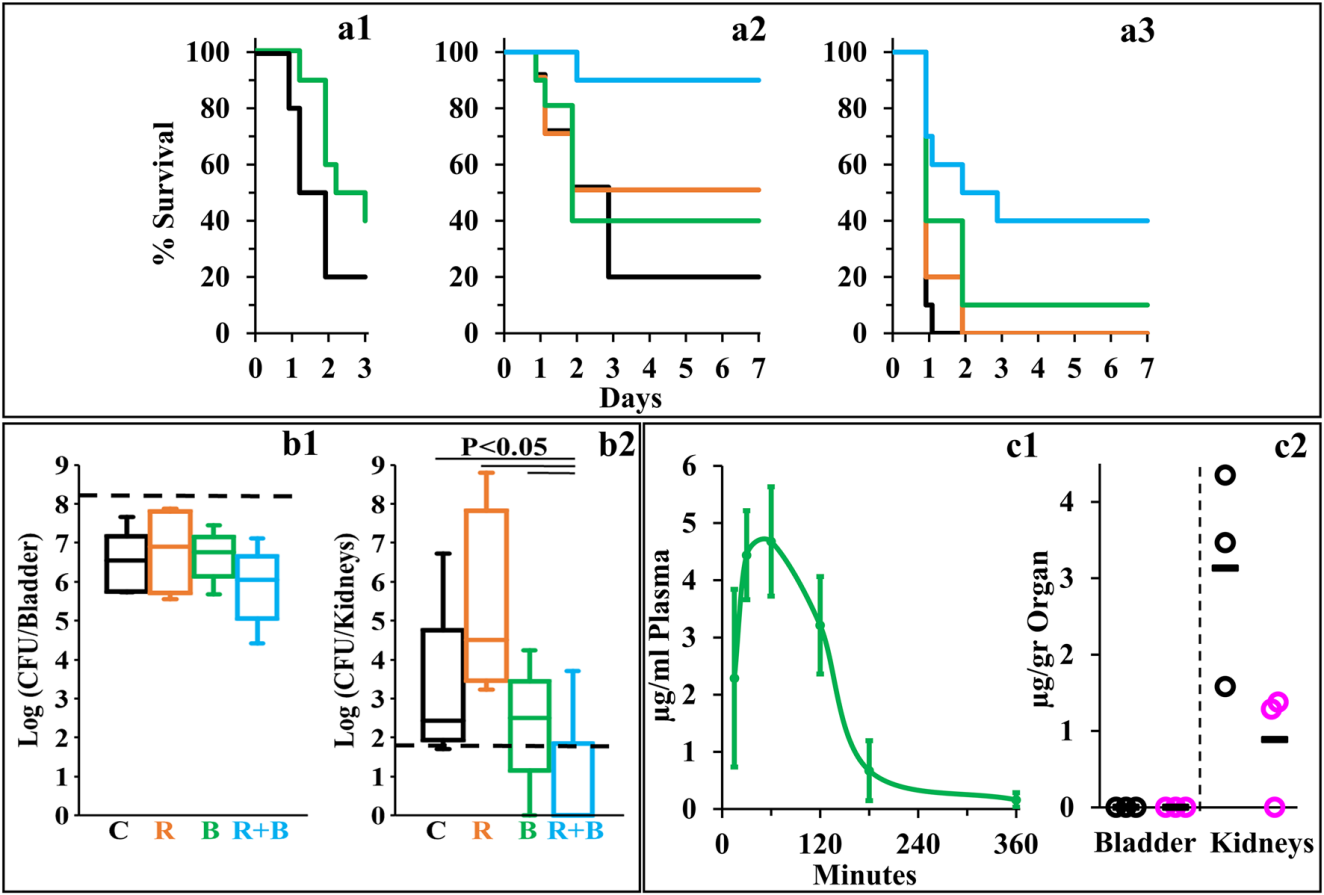
C_10_BBc_12_B Properties in Mice Models. (**a**) Percent survival of neutropenic mice (n = 10 per group) infected with *E. coli* 25922 using the peritonitis-sepsis infection model. Panel a1 shows survival rates of mice infected with 1.4 × 10^6^ CFU/mouse using a modified model, in which bacteria were pre-treated *in-vitro* for 15 min with 10 µM C_10_BBc_12_B. Bacteria pre-treated with the vehicle control, black; bacteria pre-treated with C_10_BBc_12_B, green. Panels a2 and a3 respectively show representative survival rates of mice infected with 1.7 × 10^6^ and 1.9 × 10^6^ CFU/mouse. Vehicle treated control, black; 20 mg/kg rifampin, orange; 12.5 mg/kg C_10_BBc_12_B, green; rifampin + C_10_BBc_12_B, cyan. (**b**) Organs bacterial loads in mice (n = 5 per group) infected with 1.2 × 10^8^ CFU/mouse of UPEC CFT073 using the urinary-tract infection model. Box plots in panels b1 and b2 respectively show the CFU counts assessed 24 h post-infection for bladders and kidneys. Vehicle treated control (C) black; 2 mg/kg rifampin (R) orange; 7.5 mg/kg q.i.d. C_10_BBc_12_B (B), green; rifampin + C_10_BBc_12_B (R + B) cyan. Upper and lower dashed lines respectively represent the inoculum and the limit of detection. (**c**) Quantification of C_10_BBc_12_B in organs of interest, as determined following S.C. administration of 12.5 mg/kg to mice (n = 3 per time point). Panel c1 shows C_10_BBc_12_B circulating concentrations in mice plasma. Error bars represent standard deviations. Panel c2 shows the levels detected in mice bladder and kidneys. t = 1 h, black circles; t = 3 h, pink circles; averages, black horizontal bars.

**Figure 4:**
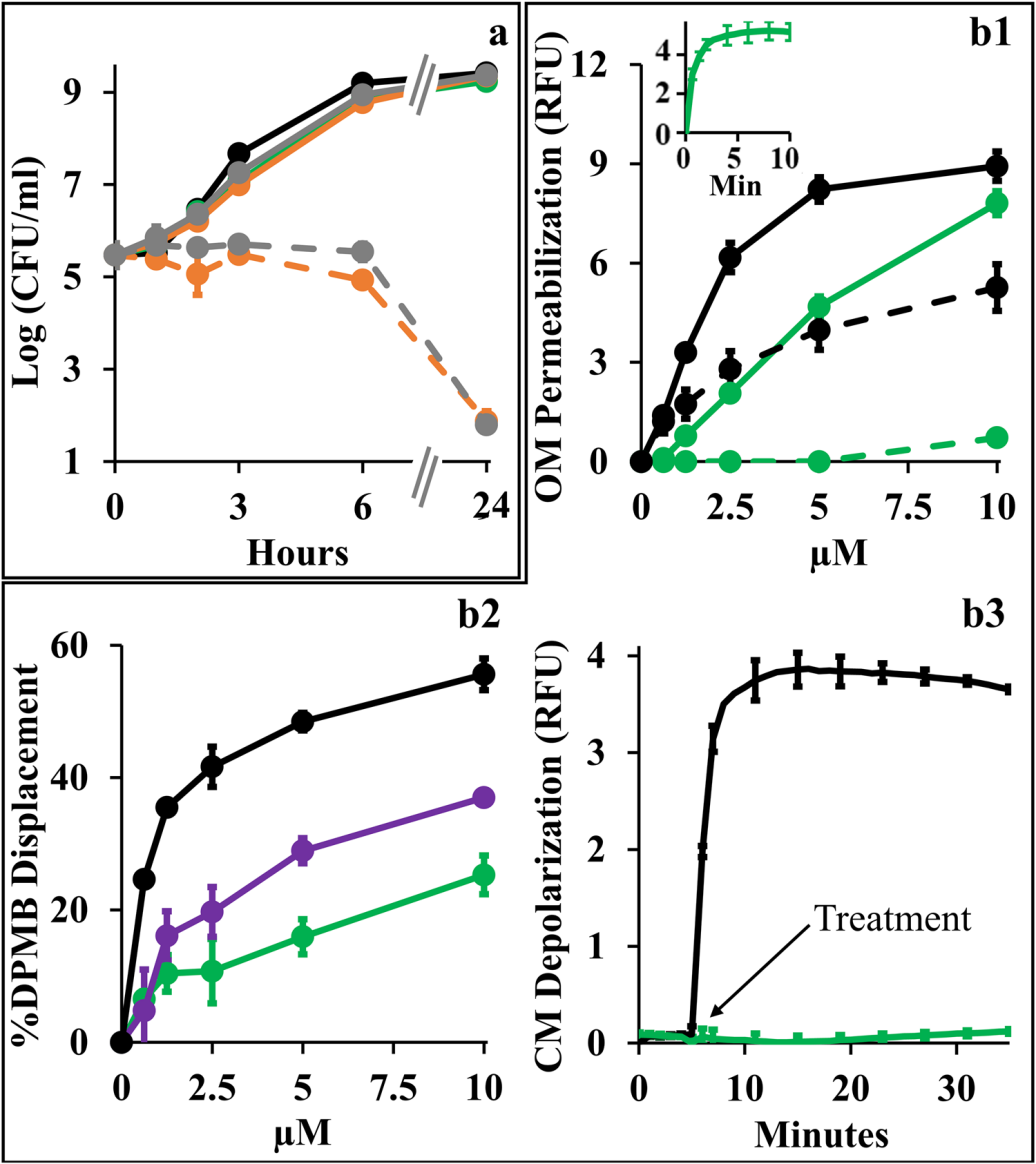
Mechanistic Studies of C_10_BBc_12_B Using *E. coli* 25922. (**a**) Growth kinetics in presence of single *versus* combined antimicrobials. Untreated control, black; 10 µM C_10_BBc_12_B, green; 1 µg/ml erythromycin, solid gray; 20 ng/ml rifampin, solid orange; 1 µg/ml erythromycin with 10 µM C_10_BBc_12_B, dashed gray; 20 ng/ml rifampin with 10 µM C_10_BBc_12_B, dashed orange. (**b1**) OM permeabilization assessed using NPN fluorescence measurements, in presence or absence of 10 mM MgCl_2_. PMB, solid black; C_10_BBc_12_B, solid green; PMB in presence of 10 mM MgCl_2_, dashed black; C_10_BBc_12_B in presence of 10 mM MgCl_2_, dashed green. Data represent fluorescence after 6 min. Inset shows representative fluorescent signal evolution kinetics for 10 µM C_10_BBc_12_B. (**b2**) Percent displacement of mono-dansylated PMB from *E. coli* LPS. PMB, black; PMBN, purple; C_10_BBc_12_B, green. Data represents results from two independent experiments. (**b3**) CM depolarization assessed using DiSC_3_(5) fluorescence measurements. 2.5 µM PMB, black; 10 µM C_10_BBc_12_B, green. Arrow denotes the moment of antimicrobials addition after DiSC_3_(5) baseline signal stabilization. RFU, relative fluorescence units. Error bars represent standard deviations.

Towards determining the potential of C_10_BBc_12_B to resolve resilient GNB infections, we used a modified peritonitis-sepsis infection model to verify whether bacteria that were exposed to the pentamer prior to administration to mice, would (in principle) be able to affect the disease course, possibly via LPS neutralization, considering that immune activation by LPS is an underlying factor for sepsis deterioration. Thus, bacteria briefly exposed to C_10_BBc_12_B (10 µM in PBS, a concentration known to affect outer-membrane (OM) permeability but does not alter bacterial proliferation rates, as will be shown in Fig. 4) were administered intraperitoneally to neutropenic mice and mortality was recorded for three days thereafter, as compared to control mice infected by the same culture pre-treated with PBS vehicle. As shown in Fig. 3a1, bacteria that were exposed to the pentamer have delayed the onset of mice mortality and reduced the overall lethality rate, thereby reflecting the pentamer’s potential ability to interfere with the sepsis process upon treating infected mice, assuming proper pharmacokinetic behavior would be achieved. Encouraged by this observation, we next prepared to assess the pentamer’s ability to affect sepsis (using the unmodified peritonitis-sepsis model) upon subcutaneous administration to infected mice of a sub-maximal tolerated dose (MTD).

Thus, MTD determination after administration of increasing doses (i.e., 0, 20 and 40 mg/kg C_10_BBc_12_B) to normal mice revealed no apparent physiological signs of distress for any of the mice, from the moment of injection throughout a seven-day monitoring period (i.e., MTD was estimated > 40 mg/kg). This represents a significant improvement compared to its analog C_10_OOc_12_O^22^, for which transient signs of distress were observed in a mouse (score of 3 out of 6) and mortality of another mouse was recorded within an hour after administering the 40 mg/kg dose (i.e., estimated MTD was > 20 but < 40 mg/kg). As the B-based pentamer’s MTD is comparable to that reported for the last-resort antibiotic PMB^40^, this finding suggests that C_10_BBc_12_B might offer an improved safety profile given its molecular attributes as small molecule and milder, non-membranolytic putative mechanism of action (as further addressed below).

Next, we evaluated the lipopeptide’s ability to affect survival of infected neutropenic mice. Figure 3a2 depicts the mice survival rates upon monotherapy or/and combination-therapies. Thus, while the control mice response to infection has evolved similarly to the experiment shown in panel a1 (*i.e.*, 80% mortality in control mice was reached at two–three days post-infection, using similar inoculum size) mice treated with C_10_BBc_12_B or rifampin displayed roughly comparable (40 and 50%, respectively) improved survival rates, as compared to the vehicle control, whereas the combination treatment reached 90% survival. The fact that C_10_BBc_12_B showed some efficacy even in absence of rifampin evokes our previous findings^22,29^ where using a similar infection model and treatment, C_10_OOc_12_O has also exhibited significant monotherapy efficacy. While the molecular basis for the pentamers’ abilities to prevent mice death in absence of an exogenous antibiotic is yet to be elucidated, we provided various lines of evidence arguing for the possible role played by one or more endogenous antimicrobials that may have substituted for rifampin’s role. If that were to be the case, one would be allowed to predict a more potent antimicrobial performance of C_10_BBc_12_B in treating infections of wild animals (as opposed to laboratory animals such as the mice used herein) since they are normally endowed with a more robust innate immunity.

Figure 3 (panel a3) provides evidence for considerable systemic efficacy even under harsher infection conditions (i.e., when mice were infected with a higher inoculum size) where lethality of control mice has reached 100% already on day one and mice treated with C_10_BBc_12_B or rifampin displayed virtually no efficacy. Under these conditions, the combination treatment managed to increase the survival rates from 0 to 40%, thus implying a synergistic outcome, reminiscent of the synergy observed in terms of SF values (Fig. 2a, b).

Next, we assessed the pentamer’s ability to affect the course of another disease, using the urinary tract infection (UTI) model, where, uro-pathogenic *E. coli* bacteria were administered by intra-urethral route and the infected mice treated with either C_10_BBc_12_B or rifampin or with a combination thereof, and bacterial loads in bladder and kidneys were quantified 24 h post-infection. To determine efficacy, we assessed different treatment regimens, including an initial single dose of 7.5 or 12.5 mg/kg C_10_BBc_12_B administered 1 h post infection, but found these regimens to be essentially ineffective, whether administered alone or in combination with rifampin. However, administration of four doses of C_10_BBc_12_B within a 24-h period, have generated a significant efficacy upon combination with rifampin. Thus, while no reduction in bacterial load was observed in bladders with any of the tested treatments (Fig. 3b1), the combination treatment has reduced the kidneys bacterial load, as evidenced by complete bacterial eradication (or prevention of colonization altogether) in 80% of the treated mice (Fig. 3b2).

Towards establishing cause and effect relationships, we next attempted to correlate these efficacy outcomes with the circulating pentamer’s levels, in the respective tissues of interest. Figure 3c1, c2 present data summarizing the biodistribution analysis showing that C_10_BBc_12_B presence overlapped with the efficacies outlined in Fig. 3a, b, respectively. Thus, similarly to its analogs^22^, the subcutaneous administration of C_10_BBc_12_B resulted in a steep increase of its circulating concentrations (reaching its maximum after 30–60 min) followed by a shallower decline that ultimately cleared most of the lipopeptide from the bloodstream after ~ 180 min. Importantly, C_10_BBc_12_B maintained antibiotics potentiating concentrations (*i.e.*, ≥ 2.5 µg/ml) for > 2 h post-adjuvant administration. Likewise, quantification of C_10_BBc_12_B in bladder and kidneys, revealed negligible amounts in the bladder, whereas the pentamer was readily quantifiable in kidneys for at least 3 h post-inoculation. Therefore, these findings align well with those observed in Fig. 3, in the sense that treatment efficacies are correlated with the adequate pentamer’s presence.

### Antibiotics potentiation is mediated by C_10_BBc_12_B interactions with the OM

Towards better understanding the synergistic mechanism, we compared individual growth kinetics to determine contributions of each antimicrobial and assessed the type and extent of implicated membrane damages, assuming that low OM-permeability underlies the observed antibiotics inefficiencies by impeding their intracellular accumulation. Figure 4a shows that at synergistic concentrations (i.e., 10 µM C_10_BBc_12_B and 1 µg/ml erythromycin or 20 ng/ml rifampin) all individual proponents were unable to significantly alter bacterial proliferation (i.e., growth curves obtained for untreated control and individual treatments, were virtually undistinguishable) whereas upon combining treatments, bacterial proliferation was arrested for at least 6 h, followed by bacterial death levels that nearly eradicated the cultures at the 24 h endpoint. Using equivalent conditions, similar growth curves have also characterized *K. pneumoniae*, *P. aeruginosa* and *A. baumannii* (data not shown). Importantly, while sub-inhibitory concentrations of some reported antibiotic potentiators (such as C_14(ɷ5)_OOc_10_O^23^ or PMBN^41^) have transiently inhibited bacterial growth in absence of antibiotics, the fact that C_10_BBc_12_B is devoid of this attribute cements our proposed classification as adjuvant. Also, C_10_BBc_12_B combinations with erythromycin or rifampin exhibited similar behaviors—despite the antibiotics distinct modes of action, hence providing additional support to the notion that their potentiation maybe mechanistically related, where the pentamer plays a facilitator role, in both cases. To test this postulate, we next assessed the extent of OM damage using N-phenyl-1-naphthylamine (NPN) fluorescence measurements (Fig. 4). As shown in panel b1, C_10_BBc_12_B, like PMB (deemed gold-standard OM-permeabilizer)^12,42^, increased *E. coli*’s OM permeability to NPN in a dose-dependent manner, albeit in a milder fashion. Validation of these findings is provided by the observation that high concentrations of MgCl_2_ have attenuated the NPN fluorescence for both agents, correspondingly. Thus, the resulting fluorescence reduction (*i.e.*, almost complete for C_10_BBc_12_B and much less for PMB) enforces the concept that C_10_BBc_12_B and PMB differ in their affinities towards LPS. This notion was further corroborated using dansyl-PMB (DPMB) competition experiments (Fig. 4 panel b2) by confirming the inferior ability of C_10_BBc_12_B to displace the LPS-bound DPMB (the relevance of this affinity difference will be addressed while discussing Fig. 6).

Another experiment stressing mechanistic difference(s) between these lipopeptides (C_10_BBc_12_B and PMB) is provided through their ability to affect cytoplasmic membrane (CM) permeability. Unlike PMB whose bactericidal activity was associated with a rapid CM-depolarization^12^ (Fig. 4 panel b3), C_10_BBc_12_B was clearly unable to affect the trans-membrane potential (TMP) at the tested concentration range (*i.e.*, up to 10 µM). The fact that a more hydrophilic analog C_8_BBc_12_B (data not shown) displayed dose–response depolarization traces that are indistinguishable (i.e., identical to that shown in Fig. 4 panel b3), supports the notion that both derivatives are indeed devoid of some minimal attribute (hydrophobicity?) required for initiating CM-damages.

The lack of observable CM damage upon C_10_BBc_12_B interaction with GNB (panel 4b3) is consistent with the kinetics results presented in panel 4a, since if such damage was to occur, some growth delay should have been apparent, as was observed with C_14(ɷ5)_OOc_10_O^23^ and PMBN^41^ as well as with C_10_KKc_12_K^24^ and to a lesser extent with C_10_OOc_12_O^22^. These observations (i.e., experiments linking OM permeabilization by C_10_BBc_12_B to its antibiotics potentiation activity) along with the results establishing the weaker interactions of C_10_BBc_12_B with both membranes in comparison with the bactericidal PMB (panels b1–b3) hence render mechanistically puzzling its greater capacity to sensitize GNB to rifampin. Of note, the fact that the displacement profile of C_10_BBc_12_B resembled more that of PMBN than PMB (panel b2), suggests that lower LPS affinities promote stronger adjuvant properties.

### Implication of efflux machinery in antibiotics potentiation

While C_10_BBc_12_B appears to hold mechanistic attributes similar to those of polymyxins, we find that these lipopeptides are distinguishable by various aspects (besides the aforementioned differences) including susceptibility to undergo efflux. When comparatively assessed against the isogenic pair of wild-type *E. coli* Ag100 and its efflux deficient mutant Ag100a (Δ*acrAB*) a higher potency of C_10_BBc_12_B (eightfold reduction in MIC value) was observed against the mutant strain (Table 2). Similar relationships were recorded with another isogenic pair of *Salmonella enterica* serovar Typhimurium. These outcomes would sit well with the notion that C_10_BBc_12_B is an efflux substrate, very much as previously proposed for its analogs C_10_KKc_12_K^26^ and C_10_OOc_12_O^29^, unlike C_14_OOc_12_O^23^ or C_14_KKc_12_K^24^. Accordingly, the polymyxins (e.g., PMB or colistin), often described as unlikely efflux-substrates^43–45^ are in fact efficient CM-destabilizers that swiftly dissipate the TMP, thereby obstructing the proper proton-dependent function of resistance-nodulation-division (RND) efflux pumps, including the AcrAB-TolC system commonly expressed in Enterobacteriaceae.

**Table 2:** Effect of RND pumps on growth-inhibitory activity.

Compound	MIC^a^ (µM) in LB				References
	*E. coli*		*S*. Typhimurium		
	Wild-Type	Mutant	Wild-Type	Mutant	
Erythromycin	** > 50**	**10.9**	** > 50**	**6.8**	^46^
Rifampin	**19.4**	**19.4**	**19.4**	**19.4**	^46^
Polymyxin B	0.7	0.7	0.7	0.7	Current study
C_14_KKc_12_K	**6.2**	**6.2**	**3.1**	3.1	^24^
C_10_KKc_12_K	** > 50**	**6.2**	** > 50**	25	^26^
C_10_OOc_12_O	** > 50**	**6.2**	> 50	6.2	^29^
C_10_BBc_12_B	50	6.2	> 50	12.5	Current study

^a^Minimal inhibitory concentrations determined using the broth microdilution method, as respectively assessed against wild-type (Ag100 and 14028) and efflux deficient (Ag100a and 14028Δ*AcrAB*) isogenic strains of *E. coli* and *S*. Typhimurium. Published data appear in bold fonts.

Thus, although both PMBN and C_10_BBc_12_B may act as antibiotic adjuvants by increasing OM-permeability, we find that they differ in relation to how they affect the TMP and how they are affected by efflux: the former causes TMP dissipation, possibly inactivating proton-dependent efflux (including of itself)^47^ whereas C_10_BBc_12_B appears relatively reluctant to interact with the CM despite its hydrophobic and cationic attributes, thereby sustaining *de-facto,* its availability for efflux from the periplasm or from superficial adhesion to the CM (as proposed for C_10_KKc_12_K^26^ but not C_14_KKc_12_K^24^).

**Figure 5:**
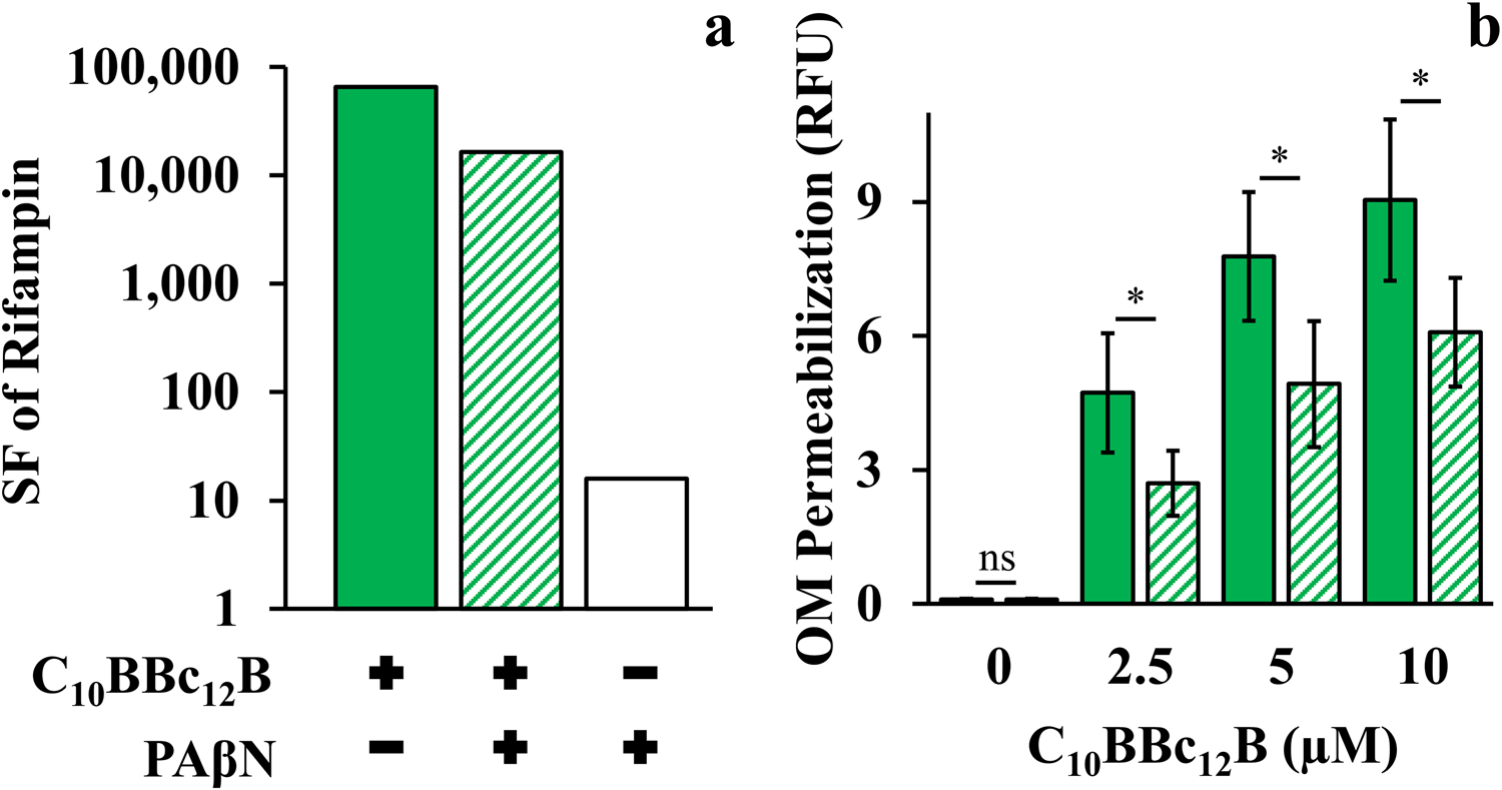
Efflux Role in C_10_BBc_12_B OM-Permeabilization Using *E. coli* 25922. (**a**) Sensitization factors (SF) determined for rifampin in presence of 10 µM C_10_BBc_12_B, solid green bars; 10 µM C_10_BBc_12_B + 5 µg/ml PAβN, striped green bars; 5 µg/ml PAβN, white bars. (**b**) OM permeabilization assessed using a modified NPN assay, following 5 min incubation with 100 µM CCCP. C_10_BBc_12_B, solid green bars, C_10_BBc_12_B in presence of 100 µM CCCP, striped green bars. Data represent fluorescence after 1 min. Asterisks denote *P* < 0.05; ns, not significant. RFU, relative fluorescence units. Error bars represent standard deviations.

Another distinguishing aspect emanated from the lipopeptides divergent behavior in presence of efflux pump inhibitors (EPIs). Since PMBN was reported to synergize with EPIs in potentiating antibiotics activity^41^, we verified whether C_10_BBc_12_B potentiation could be similarly enhanced in presence of EPIs especially since, in the pentamer’s case, efflux function stands presumably unaffected. However, introduction of EPIs appears to rather dampen the pentamer’s capacity for antibiotics potentiation. Thus, towards isolating relationships between C_10_BBc_12_B, EPI and efflux machinery, we used rifampin (instead of erythromycin) owing to its non-susceptibility for efflux by RND pumps^48^ (and in Table 2). Figure 5a shows that C_10_BBc_12_B SF values, as determined in absence of the EPI phenylalanine-arginine β-naphthylamide (PAβN)^49,50^, were in fact greater than those obtained in its presence, thereby suggesting an antagonistic relationship between C_10_BBc_12_B and PAβN or even another EPI^49,51^, unlike PMBN’s^41^. It is ought to be mentioned that while functioning as efflux pumps inhibitors, these EPIs ^52,53^ were additionally reported to increase OM permeability (as might also be inferred from our observations, as depicted in Fig. 5a). Unfortunately therefore, as this dual mechanism of action of PAβN (i.e., pump inhibitor and OM permeabilizer), complicates the assignment of either one of its functions to the antagonistic relationship with C_10_BBc_12_B, we attempted to circumvent this challenge by using an alternative approach for limiting efflux function, and depleted the fuel required for proper function of RND pumps (the proton motive force) by introducing the ionophore carbonyl cyanide m-chlorophenyl hydrazone (CCCP)^54,55^. As shown in Fig. 5b, CCCP has diminished the OM-permeabilization exerted by C_10_BBc_12_B. Importantly, NPN fluorescent signals obtained with the untreated control and with the CCCP-treated control (without lipopeptide) were practically identical, indicating that CCCP affected the proton motive force, without compromising OM integrity. These findings therefore support a scenario in which proper efflux function could boost OM-permeabilization by C_10_BBc_12_B.

### Reiterated Efflux-Propelled OM Interactions Might Boost C_10_BBc_12_B Potency

**Figure 6:**
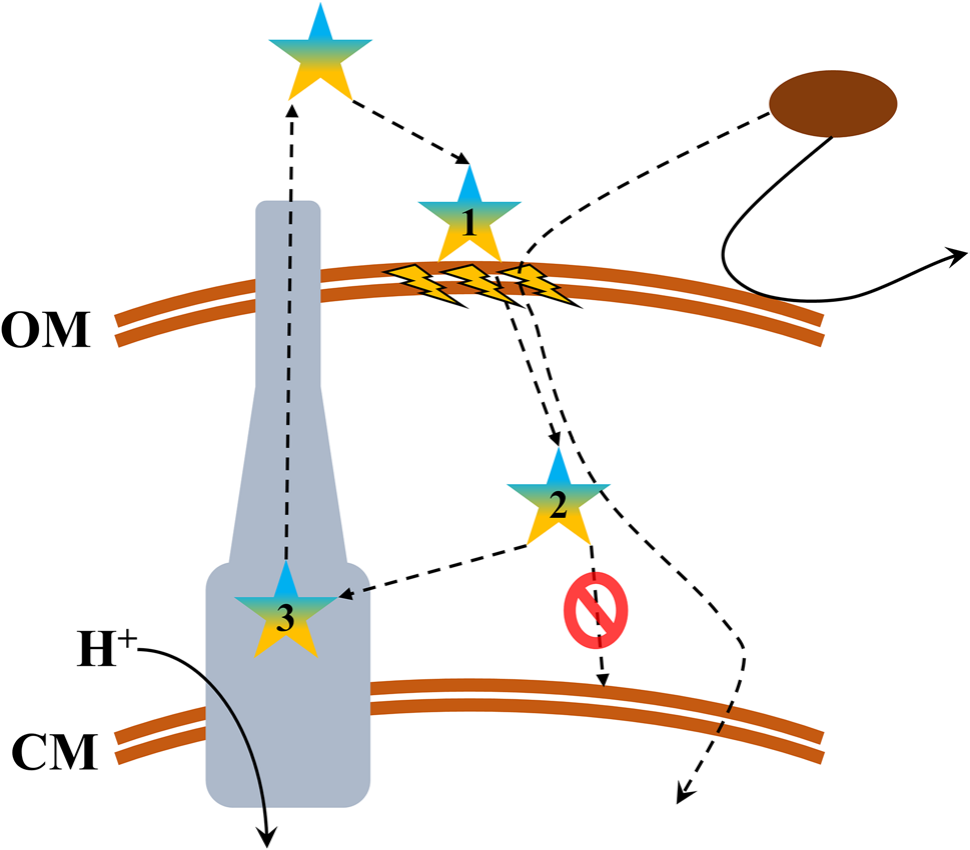
Proposed Mechanism for GNB-Sensitization to Antibiotics by C_10_BBc_12_B. The lipopeptide (pentameric star) is initially electrostatically attracted to the outer-membrane (OM) outer-leaflet, namely LPS. Upon its adhesion **(1)** C_10_BBc_12_B displaces divalent cations, thereby destabilizing the organized membrane structure and mediating cracks formation (lightning symbols) that promote the increased permeability of otherwise restricted hydrophobic antibiotics (brown oval), which are now able to translocate across the cytoplasmic-membrane (CM) and interact with their cytoplasmic targets. Likewise, C_10_BBc_12_B translocates into the periplasm **(2)**, where it experiences attraction from two opposing forces: CM and efflux machinery (gray complex, representing RND pumps). C_10_BBc_12_B affinity towards the pump is sufficiently higher to promote its expulsion **(3)** rather than its interaction with the CM (red “not allowed” symbol). Therefore, C_10_BBc_12_B efflux *de-facto* reintroduces the pentamer molecules to its site of action, the OM vicinity, thereby enabling the scenario to reiterate itself.

The data collected so far (particularly observations detailed in Figs. 4 and 5) insinuate mechanistic aspects that might explain the extraordinary potentiation capacities of C_10_BBc_12_B. In our understanding, they could be rationalized to reflect a cyclic scenario consisting of three main steps, as depicted in Fig. 6.

1. *Adhesion*: As described for PMB by the self-promoted uptake theory^56^, electrostatic attraction would initially lead to C_10_BBc_12_B adherence to anionic OM components (namely LPS). This interaction effectively displaces LPS-bound bivalent cations (that normally limit lateral motion by “gluing” adjacent lipid-A molecules) thereby expanding the monolayer fluidity. This effect is likely exacerbated by the lipopeptide’s larger molecular volume that further increases the intermolecular distances, thereby leading to transient crack formations, through which hydrophobic molecules may sift inwards.
2. *Translocation*: These presumably short bursts of membrane disruption events could nevertheless eventually mount to the translocation of otherwise excluded hydrophobic compounds (exemplified in this work by rifampin and erythromycin) thereby facilitating their interaction with cytoplasmic targets. Concomitantly, this resultant OM-permeabilization also promotes the translocation of additional C_10_BBc_12_B molecules.
3. *Expulsion*: After translocating into the periplasm, the pentamer’s fate can theoretically follow several routes, including exit the periplasm autonomously (unlikely, since it would require diffusion opposing the lipopeptide’s concentration gradient), interaction with periplasmic constituents including the CM outer leaflet and/or expulsion by efflux. Likely therefore, at this stage, C_10_BBc_12_B would be simultaneously attracted to both the CM anionic phospholipids and to the efflux pump hydrophobic pocket^57–59^. Here too, the lipopeptide’s moderate hydrophobicity might serve as a key determinant for the subsequent outcome, as it was shown that hydrophobic analogs (such as C_14_KKc_12_K^24^) might escape extrusion by efflux pumps via strong interactions and deep insertion within the CM^60,61^, where they would instigate damages to various extents^24,62^. In this respect, the pentamer’s hydrophobicity appears to be sufficiently low so as to restrict itself to a superficial CM-adherence at most, which would limit its escape from efflux (or at least prolong its unbound state in the periplasm, which increases its chances for expulsion). Notably, the intact TMP sustains the pump’s ability to expel the pentamer, which now would be able to re-adhere to the OM and repeat the cycle all over again.

To our knowledge, the finding of an adjuvant whose activity is enhanced—rather than repressed—after efflux, is hitherto unreported. One might ask, at this point: If the K-, O and B-based analogs are efflux substrates (as implied by MIC data, Table 2), how come only C_10_BBc_12_B is positively affected by efflux? A plausible reason is suggested by comparing prior studies with current data indicating that C_10_BBc_12_B may lack enough binding affinity to induce even partial CM-depolarization (Fig. 4b3) whereas using the very same assessment tools, C_10_OOc_12_O and C_10_KKc_12_K were found to interact with the CM to the point of inducing its depolarization^22,29^. Based on these observations, one might additionally speculate that transition from K to B has led to a gradual gain in OM-interacting capacity (associated with bacterial sensitization potency) while gradually losing CM-interacting capacity (associated with growth-inhibitory potency).

Moreover, beyond re-introduction to its site of action (i.e., the OM) the pentamer’s expulsion might bear another logical consequence, which is to mitigate the pumps ability to extrude other substrates (erythromycin for example). While susceptibility to efflux is *a priory* considered as inherently disadvantageous for a drug’s potency, this specific case suggests otherwise. Indeed, many antimicrobial peptides act (solely or partly) by interacting with the CM or the cytoplasm, whereas C_10_BBc_12_B seems to act exclusively on the OM—therefore its efflux actually brings the pentamer closer to its target rather than distancing it. This notion, while maybe counterintuitive, suggests that adjuvants acting as OM-permeabilizers may potentially benefit from their own efflux by effectively hijacking this resistance mechanism. In other words, susceptibility to efflux might be henceforth regarded as desirable rather than detrimental for such adjuvants, for instance during drug-screening processes or repurposing schemes.

In conclusion, this study presented several novel intriguing findings supporting the unanticipated notion that antibiotic adjuvants targeting the outer-membrane may benefit from their susceptibility to efflux and may achieve unprecedented capacities for potentiating antibiotics. The data also provide evidence supporting a novel efflux-hijacking scenario that enhances the adjuvant’s potency.

Moreover, compared with its close analog C_10_OOc_12_O, administration of C_10_BBc_12_B to mice was both better tolerated and enabled a higher degree of protection from lethal sepsis. These remarkable feats were realized despite a lower circulating concentration, a drawback overcome thanks to its higher capacity for sensitizing GNB to hydrophobic antibiotics.

## Methods

### Synthesis

Lipopeptides were synthesized in-house following standard solid-phase peptide synthesis (SPPS)^63^ procedures, employing 9-fluorenylmethyloxycarbonyl (Fmoc) chemistry^64^ (Applied Biosystems 433A Peptide Synthesizer; Foster City, CA, USA). Linear analogs were synthesized on rink-amide 4-methylbenzhydrylamine (MBHA) resins, followed by cleavage and removal of BOC protecting groups using 95% trifluoro-acetic acid in Milli-Q water. For synthesis of cyclic analogs, linear lipopeptides were first synthesized on 2-CT resins, where the N-terminal acyl was substituted with an aminated analog. These lipopeptides were cleaved from the resin using acetic acid:DCM:methanol (5:4:1). Head-to-tail cyclization took place in solution under high dilution conditions, followed by removal of BOC protecting groups as previously described. Lipopeptides were then purified to > 95% homogeneity by reverse-phase high performance liquid chromatography using a C_18_ column, employing an increasing linear gradient (1%/min) of acetonitrile in Milli-Q water—both containing 0.1% trifluoro-acetic acid. Peak identities were determined by electrospray-ionization mass-spectrometry. Purified lipopeptides were lyophilized and stored at −20 °C until use.

### Bacterial strains and culture conditions

Bacteria used in this study consisted of standard American Type Culture Collection (ATCC; Biological Industries, Beit Haemek, Israel) strains, clinically isolated (CI; Sourasky Medical Center, Tel Aviv, Israel) strains, as well as isogenic mutant strains. Gram-negative bacteria included *Acinetobacter baumannii* ATCC 19606; *Escherichia coli* ATCC 25922, 35218, CFT073, CI 14384, 16327, and the isogenic pair Ag100 (wild-type) and its efflux deficient mutant Ag100a (Δ*acrAB*); *Klebsiella pneumoniae* CI 1287, 224; *Pseudomonas aeruginosa* ATCC 27853, CI 1278, 8634; *Salmonella enterica* serovar Typhimurium ATCC 14028 (wild-type) and its isogenic efflux deficient mutant Δ*acrAB*. Bacteria were grown in Luria–Bertani broth (LB; 5 g/l NaCl, 5 g/l yeast extract, 10 g/l tryptone; pH = 7.0) at 37 °C with shaking. Over-night cultures were diluted 10-folds into fresh growth-medium and incubated at 37 °C with shaking until reaching mid-logarithmic phase. Then, bacterial concentrations were adjusted using O.D._600 nm_ measurements, and bacteria were subjected to the experimental procedures listed herein.

### Cell lines and culture conditions

Cell lines used in this study included murine RAW 264.7 macrophages and human embryonic kidney HEK-293FT cells (ATCC, Biological Industries, Beit Haemek, Israel). Cells were grown in Dulbecco’s modified eagle medium (DMEM) containing 4.5 mg/L glucose, L-glutamine, sodium bicarbonate and sodium pyruvate, supplemented with 10% fetal bovine serum and pen-strep, at 37 °C and 5% CO_2_.

### Self-assembly

Organization in solution was evaluated using static light-scattering measurements (Horiba Fluorolog-3, Jobin–Yvon; Minami Ward, Kyoto, Japan). Lipopeptides were serially twofold diluted in PBS (10 mM phosphate buffer, 154 mM NaCl; pH = 7.4), and incubated for 2 h at room-temperature. Light-scattering at a 90° angle was then recorded, while holding both excitation and emission wavelengths at 400 nm (slit width of 1 nm). The critical aggregation concentration (CAC) was extracted from the intersection of trend-lines generated for free and aggregated lipopeptide forms, on plots correlating lipopeptide concentrations and scattering intensities^65^.

### Hemolysis

Permeabilization of red blood cells (RBC) was evaluated using absorbance measurements of extracellular hemoglobin. Serial twofold dilutions of lipopeptides were incubated with 1% hematocrit of murine RBC for 3 h at 37 °C with shaking. Samples were then centrifuged at 20,000 RCF for 5 min, and absorbance of the supernatants was measured at 450 nm (BioTek Instruments Synergy HT; Winooski, VT, USA). Percent hemolysis was then calculated by (A_S_ − A_N_)/(A_P_ − A_N_) × 100; where A_S_, A_N_, and A_P_ represent the absorbance of the sample, negative control (PBS) and positive control (Milli-Q water), respectively. LC_50_ values were extracted from trend-lines generated for linear-regions of plots correlating lipopeptide concentrations and percent hemolysis.

### Minimal inhibitory concentration (MIC)

MIC was determined according to the broth microdilution method: antimicrobials were subjected to serial twofold dilutions in LB medium, and incubated with 5 × 10^5^ CFU/ml of bacteria (final volume of 200 µl) for 18–24 h at 37 °C. Then, O.D._620 nm_ was measured (BioTek Instruments Synergy HT; Winooski, VT, USA), and the MIC was defined as the lowest antimicrobial concentration for which no increase in O.D._620 nm_ was evident, compared to the untreated control.

### Cytotoxicity

Lipopeptides cytotoxicity was determined using fluorescent measurements of reduced resazurin. 2 × 10^4^ cells were first cultured in 96-well plates over-night (final volume of 100 µl) at 37 °C and 5% CO_2_. Then, the medium was removed and cells were incubated for an additional 24 h with serial twofold dilutions of the lipopeptides in growth medium. Then, 10 µl of Alamar blue were added and plates were incubated for 3 h, after which fluorescence (excitation: 530 nm; emission: 590 nm) of the reduced resazurin was recorded (BioTek Instruments Synergy HT; Winooski, VT, USA).

### Combination with antibiotics

Lipopeptides and antibiotics combinations were assessed using a modified version of the checkerboard titration method: Antibiotics MIC was determined in LB medium containing sub-inhibitory lipopeptide concentrations. The sensitization factor (SF)^46^ was then calculated by SF = MIC_A_/MIC_C_, where “MIC_A_” is the antibiotic MIC in absence of an adjuvant and “MIC_C_ “ is the antibiotic MIC in presence of an adjuvant, for a specified adjuvant concentration. Combinations were classified as synergistic when antibiotics MIC were decreased by > 4 folds. Alternatively, the fractional inhibitory concentration index (FICI) was calculated by FICI = MIC_C1_/MIC_A1_ + MIC_C2_/MIC_A2_, where “MIC_C1_” and “MIC_C2_” respectively specify the antibiotic and the adjuvant MIC values upon their combination, whereas “MIC_A1_” and “MIC_A2_” respectively specify the antibiotic and the adjuvant MIC values when tested individually. Combinations were classified as synergistic for FICI < 0.5.

### Selective pressure

The likelihood of antimicrobial-resistance development was assessed by recording MIC changes upon consecutive rounds of antimicrobials application, as in the MIC assay. Following each round, bacteria from wells corresponding to ½ of the resulting MIC were regrown in fresh media to mid-logarithmic phase and subjected to the same procedure for 15 consecutive days^66^.

### Time-kill kinetics

Antimicrobials mode of action was determined according to the rate of bacterial killing. Varying concentrations of lipopeptides, antibiotics and combinations thereof were mixed in LB medium with 5 × 10^5^ CFU/ml of bacteria (final volume of 1 ml) and incubated at 37 °C with shaking. At the designated time-points aliquots were serially tenfold diluted in saline, plated on LB agar, and incubated over-night at 37 °C for enumeration.

### Outer membrane permeabilization

Lipopeptides ability to permeabilize the OM was evaluated using fluorescent measurements of N-phenyl-1-naphthylamine (NPN)^67^. 200 µl of an over-night culture were diluted into 10 ml of fresh LB medium and incubated at 37 °C with shaking until reaching O.D._600 nm_ = 0.5. The culture was then centrifuged at 10,000 RCF for 3 min, the supernatant aspirated, and the pellet resuspended in equal volume of 5 mM HEPES containing 5 mM glucose (pH = 7.2). This suspension was then mixed with NPN (5 mM in acetone) to reach a final NPN concentration of 10 µM. Then, 190 µl of this mixture were dispensed into black microtiter plates, mixed with 10 µl of HEPES containing serial twofold dilutions of lipopeptides and fluorescence (excitation: 360 nm; emission: 460 nm) was recorded immediately for up to 10 min at 37 °C with shaking^29^ (BioTek Instruments Synergy HT; Winooski, VT, USA). In a modified version of this assay, in which efflux was inhibited, bacteria (similarly prepared) were incubated with the lipopeptides in presence or absence of 100 µM CCCP for 5 min. Samples were then twice centrifuged at 10,000 RCF for 3 min, the supernatant aspirated, and the pellet resuspended in equal volume of 5 mM HEPES containing 20 mM glucose (pH = 7.2), so as to re-energize the efflux machinery. NPN was then added (final concentration of 10 µM) and fluorescence was recorded immediately thereafter.

### Binding to lipopolysaccharide

Affinity to lipopolysaccharide (LPS) was assessed using fluorescent measurements of displaced mono-dansylated polymyxin B (DPMB)^68^. 180 µl of 5 mM HEPES containing 2 µM DPMB and 3 µg/ml LPS (from *E. coli*) were dispensed into black microtiter plates and mixed with 20 µl of HEPES containing serial twofold dilutions of lipopeptides. Plates were incubated in the dark for 1.5 h at room-temperature and fluorescence (excitation: 340 nm; emission: 485 nm) was recorded immediately thereafter (BioTek Instruments Synergy HT; Winooski, VT, USA).

### Cytoplasmic membrane depolarization

Lipopeptides aptitude to depolarize the CM was evaluated using fluorescent measurements of the transmembrane potential sensitive dye 3,3′-Dipropylthiadicarbocyanine iodide (DiSC_3_(5))^69^. Bacteria (O.D._600 nm_ = 0.1) were washed by centrifugation at 10,000 RCF for 5 min, supernatant aspiration, and pellet resuspension in 5 mM HEPES containing 20 mM glucose, 50 mM KCl and 0.2 mM EDTA (pH = 7.2). This suspension was mixed with DiSC_3_(5) (400 µM in DMSO) to reach a final DiSC_3_(5) concentration of 4 µM and was incubated in the dark for 1 h at 37 °C with shaking. Then, 180 µl of this mixture were dispensed into black microtiter plates and fluorescence was recorded (excitation: 622 nm; emission: 670 nm) at 37 °C with shaking until baseline stabilization (BioTek Instruments Synergy HT; Winooski, VT, USA). Finally, 20 µl of HEPES containing serial twofold dilutions of lipopeptides were added and fluorescence was recorded for up to 30 min at the same conditions^23^.

### *In-vivo* studies

Animal procedures were reviewed and approved by the Institutional Animal Care and Use Committee (ethics no. IL-0800519, IL-0760711, IL-0550618, IL-0320320, IL-1270821) and performed in accordance with the “Guide for the Care and Use of Laboratory Animals”, published by the US National Research Council^70^. The study is reported in accordance with ARRIVE guidelines. All protocols employed 4–5-week-old male ICR mice, weighing 20–25 g (Envigo; Jerusalem, Israel). Mice were kept on a 12 h light/dark cycle and provided with food and water *ad-libitum*. At the designated experimental end-points mice were euthanized by means of CO_2_ asphyxiation. Unless otherwise specified, all administered solutions were freshly prepared in sterile PBS immediately before administration.

### Acute toxicity

The maximal tolerated dose (MTD) was determined after sub-cutaneous (S.C.) administration of lipopeptides solutions (0.2 ml). Mice (N = 3 per group) were monitored for up to 7 days and their physiological condition was scored on a scale of 1 to 6, in which a score of 1 indicates no apparent signs of distress, and a score of 6 signifies severe distress necessitating the mice immediate euthanasia.

### Biodistribution

To assess lipopeptides circulating concentrations, mice (N = 3 per group) were S.C. administered with lipopeptides solutions (0.2 ml), sacrificed at specified times afterwards and their blood was aseptically collected. Blood samples were centrifuged at 6,000 RCF for 2 min, and 0.2 ml of the supernatant plasma were mixed with 0.5 ml of acetonitrile:methanol (1:1). This mixture was shaken for 30 min, and then centrifuged at 10,000 RCF for 10 min. 0.5 ml of the supernatant were diluted 2-folds with Milli-Q water and analyzed by UPLC-MS using a C_18_ column. Quantification was performed using a calibration-curve generated with similarly processed blood samples, initially spiked with known lipopeptide concentrations. Accumulation in organs (bladder and kidneys) was similarly determined, but was preceded with organs suspension in 5 ml of sterile PBS and homogenization for 2 min using a bench-top homogenizer, after which 0.5 ml of each sample were mixed with 1.5 ml of acetonitrile:methanol (1:1). Quantification was performed using a calibration-curve generated with fresh lipopeptide solutions of known concentrations prepared in Milli-Q water^23^.

### Efficacy

Lipopeptides potential application in monotherapy and/or combination-therapies was explored with two infection models. In the peritonitis-sepsis model^71^, mice (n = 10 per group) were first rendered neutropenic by two intraperitoneal (I.P.) injections of 0.3 ml containing 150 mg/kg and 100 mg/kg cyclophosphamide, administered 4 days and 1 day before the experiment, respectively. Mice were then infected I.P. with 1.7 × 10^6^ or 1.9 × 10^6^ CFU/mouse (0.3 ml), and treated with the vehicle control (S.C., 1 h post-infection), 20 mg/kg rifampin (oral gavage, immediately after infection), 12.5 mg/kg lipopeptides (S.C., 1 h post-infection), or both. Mice survival was recorded for up to 7 days. In a variant assay, bacteria in PBS (1.4 × 10^6^) were pre-treated with 10 µM lipopeptides for 15 min, similarly administered, and mice survival was recorded for up to 3 days. In the urinary-tract infection (UTI) model^72^, mice (N = 5 mice per group) were anesthetized by a single I.P. injection (0.2 ml) containing a mixture of ketamine (100 mg/kg) and xylazine (5 mg/kg). Following application of an analgesic 2% lidocaine gel on their penises, mice were infected intra-urethraly using a 24-gauge catheter with 1.2 × 10^8^ CFU/mouse (0.05 ml) and treated with the vehicle control (S.C., 1 h post-infection), 2 mg/kg rifampin (oral gavage, immediately before anesthesia), 7.5 mg/kg q.i.d. lipopeptides (S.C., starting 1 h post-infection), or both. Mice were sacrificed 24 h post-infection, their bladder and kidneys aseptically excised, suspended in 5 ml of sterile PBS, homogenized for 3 min using a bench-top homogenizer, serially tenfold diluted in saline, plated on LB agar, and incubated over-night at 37 °C for enumeration.

### Statistics

Unless otherwise specified, *in-vitro* data were obtained from three independent experiments performed in duplicate. Statistical analysis was performed using one-tailed t-test, assuming equal variance. The UTI assay was analyzed using Kruskal–Wallis analysis followed by the Mann–Whitney U test with Tukey’s fence of k = 3.

### Institutional review board statement

The study was conducted according to the guidelines of the Technion Animal Care and Use committee that approved all procedures, care, and handling of animals. Ethics approval codes: IL-0800519, IL-0760711, IL-0550618, IL-0320320, IL-1270821. The study is reported in accordance with ARRIVE guidelines. Mice were obtained from Envigo; Jerusalem, Israel.

## Data Availability

All data generated or analyzed during this study are included in this published article.
